# Imaging Flies by Fluorescence Microscopy: Principles, Technologies, and Applications

**DOI:** 10.1534/genetics.118.300227

**Published:** 2018-11-26

**Authors:** Sebastian Dunst, Pavel Tomancak

**Affiliations:** * German Centre for the Protection of Laboratory Animals (Bf3R), German Federal Institute for Risk Assessment, 10589 Berlin, Germany; † Max Planck Institute of Molecular Cell Biology and Genetics, 01307 Dresden, Germany

**Keywords:** imaging, labeling, transgenic reporters, FlyBook

## Abstract

The development of fluorescent labels and powerful imaging technologies in the last two decades has revolutionized the field of fluorescence microscopy, which is now widely used in diverse scientific fields from biology to biomedical and materials science. Fluorescence microscopy has also become a standard technique in research laboratories working on *Drosophila melanogaster* as a model organism. Here, we review the principles of fluorescence microscopy technologies from wide-field to Super-resolution microscopy and its application in the *Drosophila* research field.

## Milestones in the History of Fluorescence Microscopy

FOR almost 300 years, since the invention of the light microscope in the 17th century, microscopists have been limited to studies of unstained cells using transmitted white light. The phenomenon that organic and inorganic substances, such as chlorophyll and vitamins, are capable of absorbing light and emitting photons at a higher wavelength, named “fluorescence” by Stokes in 1852, has brought about the era of fluorescence light microscopy. The first fluorescence microscopes were built by August Köhler (in 1904), Carl Reichert and Oskar Heimstädt (in 1911), and Carl Zeiss and Heinrich Lehmann (in 1913), and fluorescence microscopy serves researchers in all fields of life sciences to this day.

In the early days of fluorescence microscopy, researchers were limited to specimens that autofluoresce. In the 1930s, fluorescent stains to label nonfluorescing tissues were first introduced by Max Haitinger and, in the 1950s, Albert Coons and Nathan Kaplan developed a method using antibodies that are coupled to fluorescent dyes to detect antigens in tissues. The greatest leap forward in fluorescence microscopy has been the discovery of the Green Fluorescent Protein (GFP) in the jellyfish *Aequorea victoria* by Osamu Shimomura in 1962 (Shimomura *et al.* 1962). The sequencing and cloning of GFP in 1992 (Prasher *et al.* 1992), together with the generation of the first transgenic organism by Martin Chalfie 2 years later (Chalfie *et al.* 1994), enabled endogenous protein labeling in living organisms that revolutionized biological research. The engineering of GFP variants by Roger Tsien in 1994 (Heim *et al.* 1994) allowed simultaneous visualization of multiple cellular components. In 2008, these milestones in fluorescence microscopy were honored by the Nobel Committee, which awarded Osamu Shimomura, Martin Chalfie, and Roger Y. Tsien the Nobel Prize in Chemistry “for the discovery and development of the green fluorescent protein, GFP.”

Fluorescence microscopy in combination with specific labeling methods [*e.g.*, antibodies or fluorescent proteins (FPs)] enables selective visualization of the components of living matter, from molecules and organelles to cells and tissues, in both fixed and living organisms, and with high signal-to-noise ratio (SNR). Therefore, it has become the dominant tool for visualizing living systems and a standard technique in research laboratories working on *Drosophila* as a model organism.

## Principles of Fluorescence

**Figure 1:**
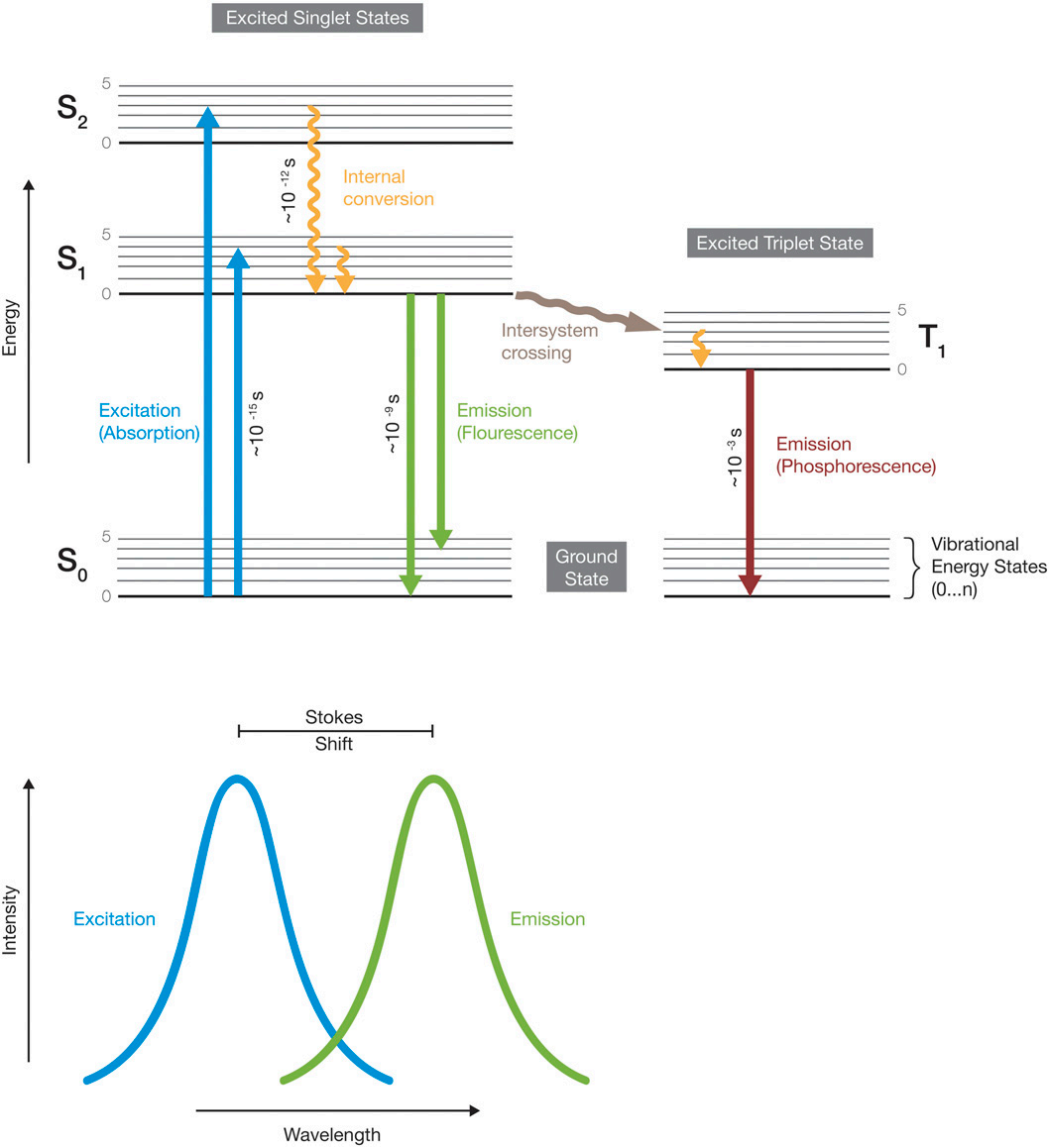
Principles of fluorescence. The Jablonski energy diagram illustrates the electronic states of a fluorophore and the transitions between them. Electronic states are indicated by horizontal lines, whereby thick lines depict the respective vibrational ground states. The transitions within and between electronic states can be nonradiative (wiggly arrows) or radiative (straight arrows), and occur at different timescales. When excited electrons return to their ground state at a nanosecond-scale, they emit fluorescence. The emission of fluorescence signals generally occurs at a longer wavelength. The shift between excitation and emission wavelengths is termed Stokes shift. S_0_, ground state; S_1_ or S_2_, higher electronic singlet energy states; T_1_, triplet energy state.

The ability of fluorescent molecules (fluorophores or fluorochromes) to absorb and emit distinct portions of light (photons) is a phenomenon referred to as photoluminescence. The relationship between the absorption and emission of light from a fluorophore is illustrated in the Jablonski energy diagram (Figure 1, top). Upon absorption of photons, a fluorophore is excited from its ground state (S_0_) to higher electronic singlet energy states (*e.g.*, S_1_ or S_2_) at the timescale of femtoseconds (Figure 1, blue). Within picoseconds, electrons in the excited state undergo nonradiative vibrational relaxation (*i.e.*, within an electronic energy state) and internal conversion (*i.e.*, between neighboring electronic energy states, Figure 1, yellow). At a nanosecond-scale, electrons return to their ground state and radiate fluorescence signals (Figure 1, green) that can be detected on a photosensitive surface. Alternatively, when excited-state electrons return to their ground state from an electronic triplet energy state (T_1_) in the millisecond-range, they emit phosphorescence signals (Figure 1, red). In biology, phosphorescence is rarely used for imaging. Rather, bioluminescent probes that generate light through a biochemical reaction, *e.g.*, firefly luciferase, are sometimes used as reporters of gene activity (Stanewsky 2007). Compared to the initial excitation wavelength, the emission of photons occurs at a longer wavelength resulting in Stokes shift (Figure 1, bottom). Fluorophores may permanently lose their ability to fluoresce due to photon-induced chemical damage and covalent modification occurring through interaction with other molecules in the long-lived excited triplet state. This phenomenon of photobleaching depends on the molecular structure of the fluorophore and the cellular environment, and can become the limiting factor, especially for live imaging studies.

## Fluorescent Labeling Techniques

Labeling techniques using fluorophores facilitate the selective visualization of biomolecules (*e.g.*, nucleic acids and proteins), dynamic cellular processes (*e.g.*, endosomal transport and signal transduction), organelles (*e.g.*, the nucleus and Golgi apparatus), behavior of single cells and cell populations (*e.g.*, cell migration and wound healing), organogenesis (*e.g.*, *Drosophila* wing and thorax morphogenesis), and even the development of entire organisms (*e.g.*, *Drosophila* embryogenesis) in fixed and living specimens.

Fluorophore-based labeling techniques are typically based on direct interactions with biomolecules (synthetic fluorescent stains and probes) or on antibody–antigen binding [immunofluorescence (IF)]. Alternatively, FPs can be genetically encoded.

### Synthetic fluorescent stains and probes

**Figure 2:**
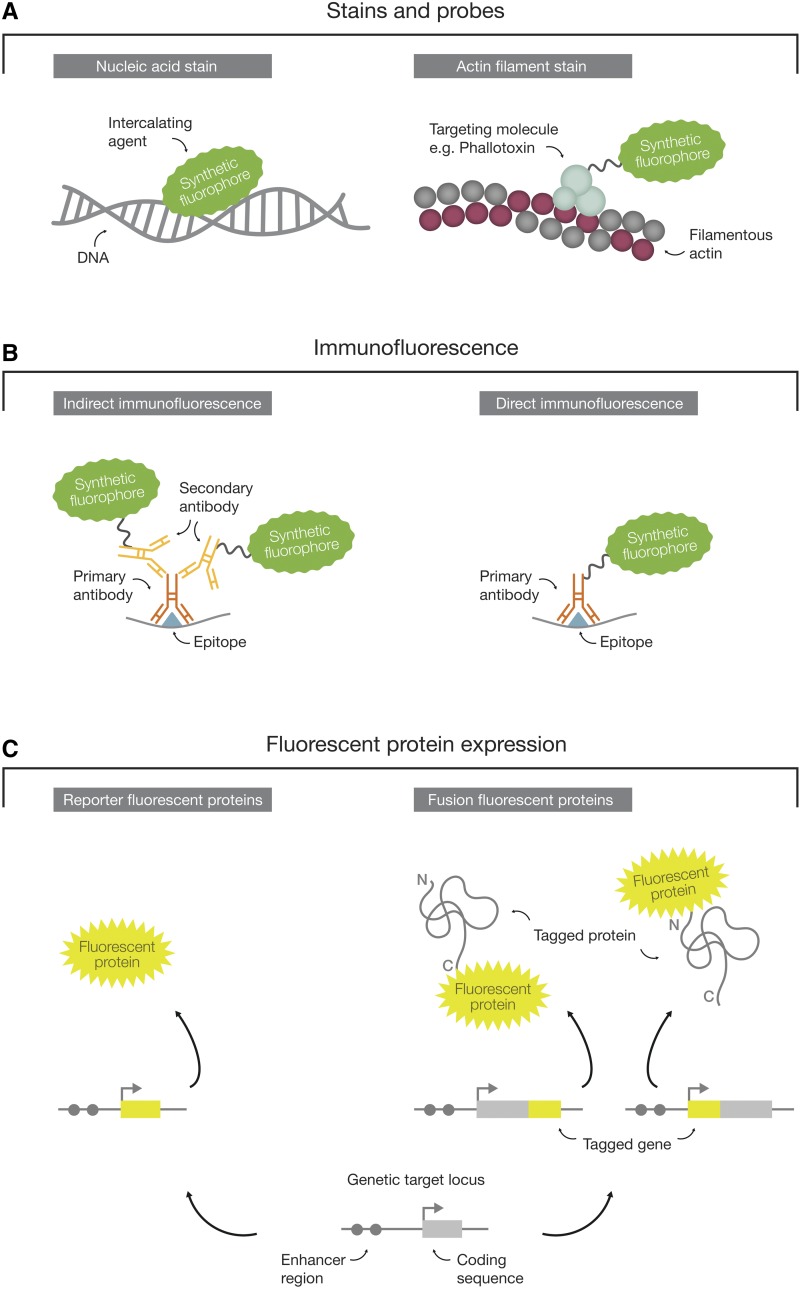
Fluorescent labeling techniques. The great diversity of synthetic fluorophores facilitates the visualization of cellular components by different fluorophore-based labeling techniques. (A) Fluorescent stains and probes directly interact with cellular components (*e.g.*, by intercalation into the DNA double helix) or may be fused to a targeting molecule (*e.g.*, phallotoxins binding to actin filaments). (B) In immunofluorescence, synthetic fluorophores are coupled to immunoglobulins (*e.g.*, IgG and IgM) that selectively bind to specific antigens of target proteins. Synthetic fluorophores may be coupled to a primary antibody that directly interacts with the target protein (direct immunofluorescence). An unlabeled primary antibody can also serve as a platform for binding of multiple secondary antibodies that are coupled to synthetic fluorophores, thereby amplifying the signal intensity (indirect immunofluorescence). (C) The endogenous expression of fluorescent proteins (FPs) provides a genetically encoded avenue for the visualization of cellular components. FPs may be expressed under the control of regulatory elements (*i.e.*, reporter FPs) or fused in-frame with the genetic sequence of a protein-coding gene to create a tagged version of the target protein (*i.e.*, fusion FPs).

Synthetic fluorescent stains and probes (Figure 2A) are typically applied to fixed cells or tissues. For live imaging approaches, cell permeability of the applied synthetic fluorescent stain needs to be considered. The most commonly used synthetic fluorescent stains and probes include reagents to selectively stain nucleic acids (*e.g.*, Hoechst 33258 and DAPI), lipids of biological membranes (*e.g.*, NileRed, FM dyes, and BODIPY), cellular structures (*e.g*., fluorophore-derivatized phallotoxins from *Amanita phalloides* to label actin filaments), and organelles (LysoTracker, MitoTracker, and ER-Tracker to label lysosomes, mitochondria, and the ER).

### Immunofluorescence

IF is an antibody-based staining technique using immunoglobulins (*e.g.*, IgG or IgM) coupled to synthetic fluorescent dyes (Figure 2B). IF allows the visualization of virtually any protein in cells or tissues. However, it is unsuitable for live imaging approaches as fixation and membrane permeabilization is required prior to staining. Selectivity of IF staining procedures is provided by the reaction of a primary antibody that has been raised against a specific antigen of interest. Each primary antibody is then targeted by multiple secondary antibodies that are coupled to a synthetic fluorescent dye (*i.e.*, indirect IF). Due to this signal amplification, IF provides superior specimen contrast. Alternatively, primary antibodies can be directly labeled with fluorophores (*i.e.*, direct IF), reducing background staining and the duration of the staining procedure. IF allows a great degree of flexibility in choosing synthetic dyes that are fluorescent at different wavelengths and can be combined with direct labeling of other cellular components. In this way, multicolor IF facilitates the simultaneous visualization of several cellular components.

### Expression of genetically encoded fluorescent proteins

The above-mentioned labeling techniques are time-consuming and are often restricted to fixed specimens. Since the game-changing discovery of GFP (Shimomura *et al.* 1962), a great variety of FPs have been engineered (Heim *et al.* 1994) that can be expressed from genetically encoded constructs. These constructs can then be used to either profile gene expression (*i.e.*, reporter FPs) or to label proteins *in vivo* (*i.e.*, fusion FPs) (Figure 2C). The expression of reporter FPs is spatially and temporally controlled by a given promoter and its regulatory elements, and hence mimics only the expression of the corresponding gene. In contrast, fusion FPs are expressed in-frame with the gene of interest providing the ability to visualize the protein product within cells. Applications of reporter FPs range from the profiling of cell type-specific gene expression patterns (see *Gene expression and protein localization patterns*) to labeling of cells for lineage tracing (see *Labeling of cells*). Fusion FPs allow, besides the visualization of subcellular localizations (see *Gene expression and protein localization patterns*), the measurement of protein dynamics (see *Protein dynamics and protein numbers*) and protein–protein interactions (see *Protein interactions*).

In *Drosophila*, these genetically encoded transgenic constructs are traditionally engineered *in vitro* and subsequently introduced into the fly genome by transgenesis. Once in the genome, the transposon-based transgenes can be mobilized by genetic techniques to insert at more-or-less random genomic locations. When inserted near gene regulatory elements, they act as reporter FPs known in *Drosophila* as gene/enhancer traps. Elaborate strategies have been developed to select fusion FPs (protein traps) that seamlessly insert the FP into the coding sequence of a gene (Venken *et al.* 2011). To date, a plethora of genetically encoded transgenic constructs carrying FPs (also LacZ and Gal4) have been introduced into the *Drosophila* genome by various transgenesis approaches. A more detailed review on gene-tagging techniques in *Drosophila* has been published in the FlyBook compendium (Kanca *et al.* 2017). The protein trap toolbox includes an extensive set of transposable elements [*e.g.*, *P* element or piggyBac transposons (Morin *et al.* 2001; Kelso *et al.* 2004; Buszczak *et al.* 2007; Quinones-Coello *et al.* 2007; Lowe *et al.* 2014), or Minos transposons (Singari *et al.* 2014; Nagarkar-Jaiswal *et al.* 2015)] that randomly integrated in close vicinity to various *Drosophila* genes. Insertions of larger genomic DNA fragments (fosmids or BACs) (Venken *et al.* 2006; Sarov *et al.* 2016) were generated by targeted transgenesis using site-specific integration (Groth *et al.* 2004). However, the most physiological way of generating protein fusions is the insertion of the FP sequence into the endogenous locus by targeted transgenesis using homologous recombination (Rong and Golic 2000; Maggert *et al.* 2008) and, more recently, clustered regularly interspaced short palindromic repeats (CRISPR)/Cas9 (Gratz *et al.* 2015). This strategy ensures endogenous expression levels of FP-tagged proteins and makes them suitable for loss-of-function studies, through targeted interference or degradation of the FP tag at the RNA [*e.g.*, GFP-RNA interference (Neumuller *et al.* 2012)] or protein level [*e.g.*, DeGradFP (Caussinus *et al.* 2011)]. However, it has to be considered that FP–protein fusions may interfere with the localization, dynamics, or function of the corresponding protein.

FPs can be also used indirectly to visualize transcription or mRNA localization (see *Labeling of nucleic acids*). While a “green fluorescent RNA” equivalent to GFP is not available in the RNA world, *Drosophila* researchers have pioneered RNA visualization techniques relying on the fusion of FPs to RNA-binding proteins (RBPs) and the engineering of recognition sequences for the RBPs into the transcripts that are to be visualized (Bertrand *et al.* 1998; Forrest and Gavis 2003). In addition, FPs can also be fused with lipid-binding motifs or protein-sorting motifs to be targeted to distinct subcellular regions (see *Labeling of organelles and other cellular structures*).

Although reporter FPs or fusion FPs can also be imaged in fixed samples, they are meant primarily for live imaging as they are constantly expressed in cells. This constant synthesis of FP pools balances excitation laser-induced photobleaching (see *Principles of Fluorescence*) and facilitates time-resolved functional studies. However, it has to be considered that weakly expressed FPs may be hard to detect and that the signal might be lost quickly due to photobleaching. Moreover, the emergence of the signal is delayed due to the time it takes for various FPs to fold before they acquire their fluorescent properties. Likewise, reporter FPs in particular may not fully reflect gene expression dynamics due to different protein degradation rates.

Conventional FPs are applicable to a broad spectrum of fluorescence microscopy technologies as described in the section entitled *Fluorescent Labeling Techniques*. Recent developments of FPs include photoactivatable, photoswitchable, and photoconvertible FPs that can be used to study protein dynamics (see *Protein dynamics and protein numbers*), or for single-molecule-based Super-resolution microscopy (see *Super-resolution microscopy*).

## Fluorescence Microscopy Technologies

Commonly used fluorescence microscopy technologies include wide-field microscopes (see *Widefield microscopy*), optical sectioning microscopes (see *Optical sectioning microscopy*), and superresolution microscopes (see *Super-resolution microscopy*) (Figure 4, A–C). Although all fluorescence microscopy technologies are generally based on the excitation of a fluorophore with a specific range (band) of wavelengths and subsequent detection of the emitted photons on a camera system, they differ by their specimen illumination and signal-detection strategies.

### Principles of sample illumination and detection

In 1893, August Köhler introduced a method, termed “Köhler illumination,” that provided optimal specimen illumination by evenly spreading the light across the entire field of view. The illumination of fluorescently labeled samples is achieved by two kinds of light sources. Arc (burner) lamps (*e.g.*, Mercury or Xenon burners) emit light at multiple wavelengths and are therefore typically used in combination with dedicated filter sets that limit the excitation spectrum to a distinct wavelength (*e.g.*, Widefield microscopy, Figure 4A). A more flexible, yet more costly, alternative is the utilization of lasers [*e.g.*, gas (argon-ion) lasers, solid-state lasers, and diode lasers] that emit light at a unique wavelength (*e.g.*, Laser scanning confocal microscopy, Figure 4B). After excitation, fluorophores emit fluorescent light at longer wavelengths (see the description of Stokes shift in *Principles of Fluorescence*), which is captured on a photosensitive surface (digital detector) to generate a digital image.

The digital detector system is a critical component of fluorescence microscopes that determines their performance and applicability. Commonly used light detectors can be classified into two categories based on the detection principle and differ in dynamic range, sensitivity, and imaging speed. Area detectors, such as the Charge-Coupled Device (CCD) and Complementary-Metal-Oxide-Semiconductor (CMOS) detector capture all emitted photons at once. The signal of an incoming photon that hits a silicon diode photosensor (commonly denoted as a pixel) generates a charge that is initially stored in a charge storage region, and, finally, is read out by an amplifier. The number of incoming photons can be regulated by adjusting the laser intensity or exposure time. However, maximal image resolution is physically constrained by the pixel dimensions on the camera chip. These types of detectors are superior for imaging applications that depend on high scan speed (*e.g.*, light sheet microscopy and spinning disk microscopy, Figure 4B).

Electronic detectors, such as photomultiplier tubes (PMTs), are typically used in microscopy technologies that scan the specimen point-by-point. For each scanned point in the sample, the emitted photons hitting the detector generate electronic charges that are individually sensed and amplified. The signal intensity can be regulated by adjusting the laser intensity, scan speed (pixel dwell time), and gain (signal amplification at the PMT). Furthermore, pixel dimensions can be flexibly adjusted. Hence, maximal image resolution is only governed by the physical properties of the light. These sensitive types of detectors are used whenever spatial image resolution is prioritized over imaging speed (*e.g.*, Laser scanning confocal microscopy, Figure 4B).

### Widefield microscopy

Since its invention in 1929, the wide-field epifluorescence microscope has become indispensable for cell biology (Ellinger and Hirt 1929; Ploem 1967). The sample is illuminated across the entire field of view and emitted fluorescence is collected on an area detector (Figure 4A). Wide-field microscopes have the advantage that a small light dose is sufficient to illuminate the specimen. Hence, imaging speed is increased, while bleaching of fluorophores and phototoxic effects on cells are reduced (Icha *et al.* 2017; Laissue *et al.* 2017). For this reason, wide-field microscopes are often used for long-term imaging of living specimens. On the other hand, this illumination/detection strategy also collects out-of-focus image information (stray light) emerging from regions outside the focal plane, compromising the resolution of image features. Stray light can be reduced by introducing a periodic grid into the illumination path (*i.e.*, structured illumination) followed by subsequent computational processing of the image that removes the mask and increases resolution (Neil *et al.* 1997). Alternatively, different postacquisition methods can be applied to improve the spatial resolution after the wide-field image has been captured (Verdaasdonk *et al.* 2014). For example, deconvolution removes stray light by applying a mathematical algorithm (Sage *et al.* 2017). However, postprocessed images always need to be compared to the original image to exclude image-processing artifacts.

### Optical sectioning microscopy

Compared to conventional wide-field epifluorescence microscopy, optical sectioning microscopes collect light only from the image plane that is in focus. Different optical sectioning-based microscopy technologies exist, among them Laser scanning confocal microscopy, multiphoton microscopy, light sheet microscopy, and spinning disk microscopy (Figure 4B).

#### Laser scanning confocal microscopy:

In a fluorescent confocal microscope setup, the laser scans the specimen point-by-point and emitted stray light from planes outside the focus is removed by a pinhole near the PMT detector (Figure 4B). The first applications of this optical slicing technology emerged in 1987 (van Meer *et al.* 1987; White *et al.* 1987) and obviated the need for tissue sectioning to image thick specimens. However, reduced signal intensity due to the elimination of stray light commonly needs to be compensated by increased laser intensity to sufficiently illuminate the specimen. In addition, the imaging laser does not only excite fluorophores in the focal plane but also all other planes along the illumination path. Hence, bleaching of fluorophores and phototoxic effects on cells might be increased.

As in most other research fields, confocal microscopy is a standard “workhorse” technology in *Drosophila* laboratories, facilitating high-resolution imaging of fixed specimens of up to 100 μm in thickness. However, due to the optical properties of light, the spatial resolution of laser-scanning confocal microscopes is limited to roughly 200 nm (see *Super-resolution microscopy* for details) preventing the separation of more densely packed structural elements in cells. Recently introduced, expansion microscopy provides an elegant means to overcome this limitation. Through the physical expansion of *Drosophila* embryos and larval and adult tissues in a swellable polymer hydrogel, this methodology has pushed the resolution limit of a standard confocal microscope to ∼70 nm (Jiang *et al.* 2018). However, its application is strictly limited to fixed specimens.

#### Spinning disk microscopy:

Spinning disk microscopy combines the advantages of laser-scanning confocal microscopes with a significantly increased acquisition speed. Instead of using single beams and pinholes, spinning disk microscopes exploit the concept of multiplexing by illuminating the sample with an array of multiple focused laser beams that scan across the specimen (Petráň *et al.* 1968) (Figure 4B). In modern spinning disk microscopes, the illuminating laser light is focused through a rotating disk harboring multiple microlenses. The emitted fluorescent signal returns along the excitation path, is cleared from stray light at a second rotating disk harboring multiple pinholes of a fixed diameter, and is finally collected on a camera system.

Due to the increased imaging speed, spinning disk microscopy reduces the bleaching of fluorophores and mitigates phototoxic effects, at the expense of slightly reduced image resolution due to the fixed pinhole diameter. Hence, this imaging technology has been employed to study cellular behaviors during fast morphogenetic events such as pupal wing development in *Drosophila* (Etournay *et al.* 2015).

#### Multiphoton microscopy:

In 1990, pulsed near-infrared lasers with longer wavelengths were used to excite fluorophores only at the plane where two excitation photons arrive simultaneously (Denk *et al.* 1990) (Figure 4B). Due to this multiphoton (or two-photon) illumination strategy, stray light emerging from out-of-focus planes is almost eliminated and a pinhole near the electronic detector is not required. Hence, only small amounts of photons are necessary to sufficiently illuminate the specimen, thereby efficiently reducing fluorophore bleaching and phototoxic effects on cells.

In addition, due to reduced light scattering at longer wavelengths, multiphoton microscopy allows higher specimen penetration depths of multiple 100 μm. Hence, multiphoton microscopy has been used for long-term four-dimensional live imaging of embryonic cell migration (Supatto *et al.* 2009) and organogenesis in intact larvae (Lin *et al.* 2008). However, due to refractive aberration, scattering, and absorption by the specimens, image resolution still diminishes with increasing imaging depth. Incorporating adaptive optics into the microscope layout [reviewed in Ji (2017)] can help to improve the quality of images from deep within tissues, which has recently been demonstrated by transcuticular imaging of the fly brain at cellular and subcellular resolution (Tao *et al.* 2017).

Multiphoton microscopy also allows label-free imaging of periodic structures such as *Drosophila* muscles or the trachea system by capturing Second Harmonic Generation signals (Lin *et al.* 2008), and water–lipid and water–protein interfaces (*e.g.*, biomembranes and extracellular matrix structures) by capturing Third Harmonic Generation signals (Débarre *et al.* 2006).

#### Light sheet microscopy:

In contrast to the above-mentioned optical sectioning microscopy technologies, in light sheet microscopy, the illumination and detection paths are perpendicular to each other (Figure 4B), a concept first introduced in 1993 (Voie *et al.* 1993). Building on this basic idea, Selective Plane Illumination Microscopy (SPIM) emerged in 2004, facilitating unprecedented imaging speeds at cellular and subcellular resolution (Huisken *et al.* 2004). In light sheet microscopy, the specimen is illuminated by a focused light sheet generated, for example, by a cylindrical lens in the illumination path. In this way, a specific plane (optical section) of the specimen is selectively and directly illuminated across the entire field of view. All emitted fluorescence signals are collected at once by fast area detectors (*e.g.*, CMOS technology) included in the perpendicularly oriented detection path of a light sheet microscope. Hence, this illumination strategy enables imaging speeds that are multiple orders of magnitude faster than any other optical sectioning microscopy technology. Due to the fact that only the imaged focal planes are illuminated at any given time, bleaching of fluorophores is strongly reduced and phototoxic effects on cells in a life-imaging setup are almost negligible (Icha *et al.* 2017; Laissue *et al.* 2017). In addition, the sample can be mounted in a way that allows its rotation around the *z*-axis to facilitate illumination and imaging from multiple angles (Schmied and Tomancak 2016). In general, light sheet microscopy often requires unconventional approaches to sample mounting, breaking away from the “biology on coverslips” paradigm (even though such an arrangement is also possible) [reviewed in Pampaloni *et al.* (2007)]. Furthermore, it has been suggested that light sheet microscopes should be built around the sample and consequently the sample can be mounted in the most physiological manner compatible with its long-term health [reviewed in Power and Huisken (2017)].

Due to its gentle optical sectioning, light sheet microscopy is commonly used in *Drosophila* research for live imaging of dynamic and/or long-term processes, such as whole-CNS functional imaging (Lemon *et al.* 2015) or embryogenesis (Khairy *et al.* 2015; Schmied and Tomancak 2016). Several extensions of the basic SPIM concept have improved image quality and acquisition speeds by implementing confocal slit detection (de Medeiros *et al.* 2015), dual-sided illumination and detection [Multiview (MuVi)-SPIM] (Krzic *et al.* 2012), high-speed simultaneous multiview (hs-SiMView) microscopy with multiphoton excitation (Lemon *et al.* 2015), and hyperspectral SPIM imaging (Jahr *et al.* 2015) . Interestingly, the open access OpenSPIM project, designed to foster broader availability of the technology, has been developed for and demonstrated on primarily *Drosophila* applications (Pitrone *et al.* 2013). However, users of any light sheet technology have to consider beforehand that the storage and processing of huge amounts of generated image data will challenge their computer hardware and software capabilities (Reynaud *et al.* 2015). Once again, elegant open source solutions to process and visualize such huge data sets have been developed specifically for *Drosophila*-related applications (Saalfeld *et al.* 2009; Preibisch *et al.* 2010; Pietzsch *et al.* 2015; Schmied *et al.* 2016).

### Super-resolution microscopy

The resolution of conventional fluorescence microscopes is fundamentally limited due to the wave nature of light and its diffraction at optical microscope components. This is reflected by two closely related physical laws: the Abbe criterion (diffraction of lines) described in 1873 (Abbe 1873) and the Rayleigh criterion (diffraction of point objects) described in 1896 (Rayleigh 1896). Both criteria define that the resolution limit, *i.e.*, the minimal distance between two objects that can still be distinguished as individual entities, depends on the wavelength and the collection angle of light that enters the objective (see below).

**Figure 3:**
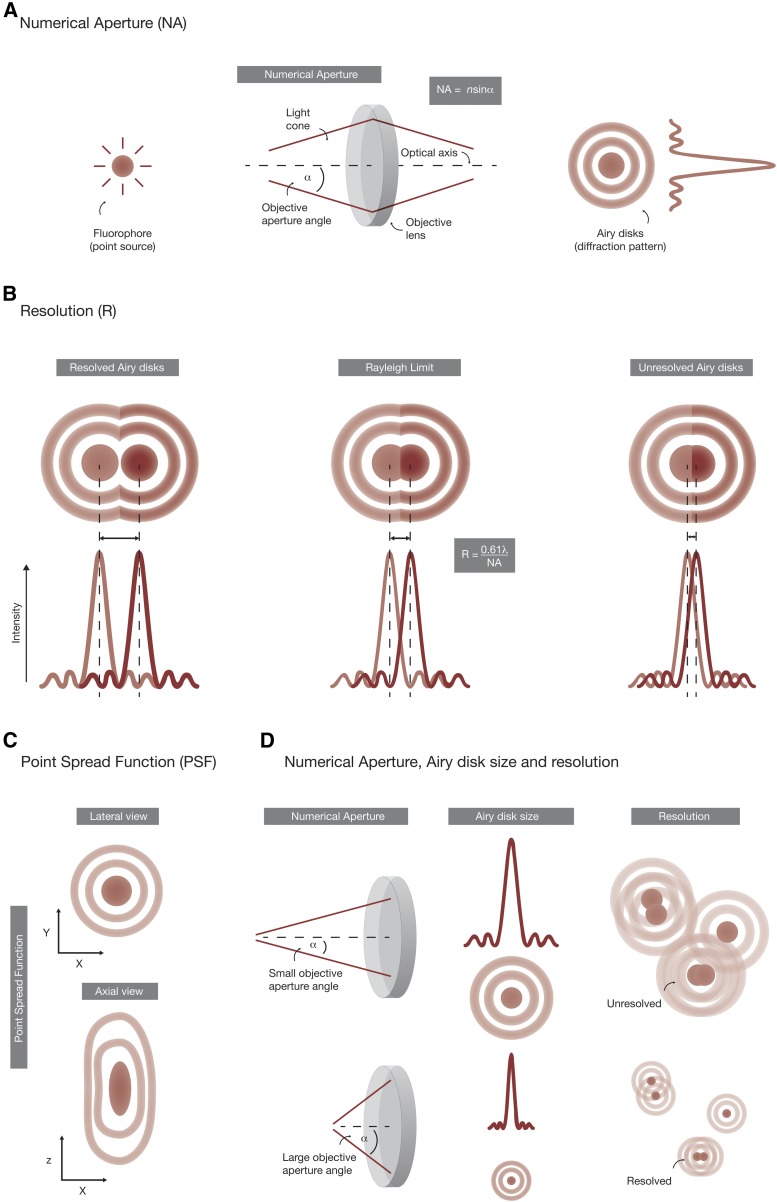
Numerical Aperture (NA), Airy disks, and image resolution. (A) Fluorophores are considered as point sources of light that emit photons. Due to the wave nature of light, emitted photons are diffracted at the optical components of the microscope and eventually appear as regularly spaced rings (Airy disks) at the detector unit. The number of photons that can be collected by the objective depends on the size of the objective aperture angle (α) and the refractive index of the sample immersion medium (n). Together, both values define the NA (NA = nsinα) of an objective, which is a dimensionless measure that describes its performance. The NA of objectives that are used for fluorescence microscopy typically ranges from 0.2 to 0.95 for air objectives and 0.85 to 1.4 for oil objectives. (B) The ability of a fluorescence microscope to resolve two point sources of light that are in close proximity is essentially defined by the Rayleigh criterion (R = 0.61λ/NA). It states that the maximum resolution of a fluorescence image is roughly half the emission wavelength of the fluorophore. When two fluorophores are further apart they can still be resolved, while fluorophores that are too close to each other appear as a single spot. (C) The Point Spread Function (PSF) is the three-dimensional representation of the Airy pattern and a unique fingerprint of each imaging system. In the lateral dimension, the PSF appears as regular rings, while in the axial dimension these rings appear as elongated ellipsoids. Hence, the shape of the PSF resembles that of an hourglass. (D) The NA of the optical system fundamentally defines its capability to resolve details in the image. Larger collection angles generate smaller Airy disks of individual fluorophores at the detector and consequently produce images with greater resolution.

In fluorescence microscopy, fluorophores are considered as point sources of light, whose emitted photons are collected by the objective and eventually projected onto the detector unit (Figure 3A). The collection angle (α) of the objective and the refractive index (n) of the sample immersion medium characterize the ability of an optical system to collect light, which is described by a dimensionless number termed the Numerical Aperture (NA). After emitted photons have passed the optical components of the microscope, their images at the detector appear as regularly spaced ring-like diffraction patterns, *i.e.*, Airy Disks, with local maxima and minima (Airy 1835). With regard to the Rayleigh criterion, two diffraction-induced Airy disks are just resolved when the central region of one Airy pattern overlaps with the first minimum of another Airy disk (Figure 3B). Hence, the minimum distance between two point objects in the lateral dimension is equal to the radius of the central Airy disk. In the axial dimension, an Airy disk has a unique elliptical pattern, termed the Point Spread Function (PSF), whose shape depends on the optical microscope components, the mounting medium, and the imaged specimen itself (Figure 3C). As a practical example, the lateral resolution limit of two GFP molecules emitting light at 510 nm would be 222 nm when using a powerful NA 1.4 microscope objective, while the axial resolution is typically reduced by a factor of roughly 2.5. Given the size of a single GFP molecule of ∼2–4 nm, hundreds of different GFP proteins may be detected as a single spot. Therefore, the selection of fluorophores emitting light at shorter wavelengths, as well as the use of an objective with a high NA, are the traditional means to improve image resolution within the limits of physical laws of diffraction (Figure 3D).

**Figure 4:**
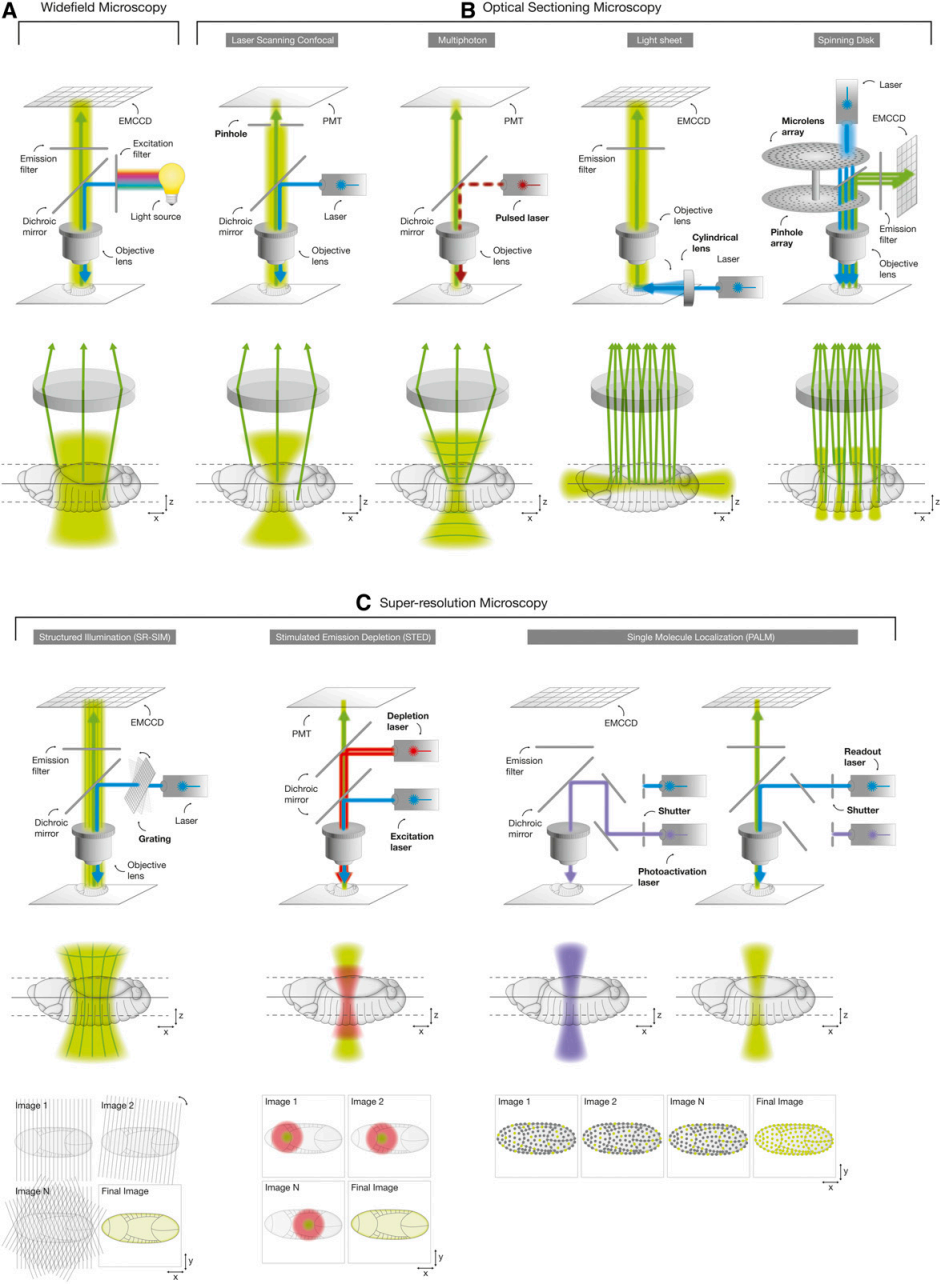
Fluorescence microscopy technologies. Fluorescence imaging technologies can be classified into three main categories, *i.e.*, Widefield microscopy (A), optical sectioning microscopy (B), and Super-resolution microscopy (C), that essentially differ in terms of the organization of the excitation (blue) and emission (green) beam path, imaging speed, and specimen invasiveness, as well as the achievable contrast and resolution of the final image. The excitation of fluorescently labeled samples is achieved by illumination with arc lamps that emit light at multiple wavelengths or lasers that emit light at a unique wavelength. The detection of emitted fluorescence signals occurs on a photosensitive surface (cameras) and electronic point detectors (PMTs). electron multiplying charge-coupled device (ECCMD); PALM, Photo-Activated Localization Microscopy; PMTs, photomultiplier tubes; SR-SIM, Superresolution Structured Illumination Microscopy; STED, STimulated Emission Depletion.

Relatively recently, the diffraction limit has been broken through the development of two fundamentally different superresolution techniques: patterned light illumination microscopy techniques, *e.g.*, STimulated Emission Depletion (STED) microscopy (Hell and Wichmann 1994) and Superresolution Structured Illumination Microscopy (SR-SIM) (Gustafsson 2000), and single-molecule localization-based microscopy techniques, *e.g.*, Photo-Activated Localization Microscopy (PALM) (Betzig *et al.* 2006; Hess *et al.* 2006) and STochastic Optical Reconstruction Microscopy (STORM) (Rust *et al.* 2006) (Figure 4C). In 2014, this pioneering work was honored with the Nobel Prize in Chemistry, which was jointly awarded to Eric Betzig, Stefan W. Hell, and William E. Moerner “for the development of superresolved fluorescence microscopy.”

#### SR-SIM:

SR-SIM is an extension of wide-field imaging microscopy that is capable of generating images with roughly double the lateral and axial resolution compared to conventional optical sectioning microscopes (Gustafsson 2000). Instead of using a uniform field of light (*i.e.*, Köhler illumination), SR-SIM is based on patterned illumination generated by a periodic grid that translates and rotates in the illumination path during the imaging procedure (Figure 4C). The interference pattern (*i.e.*, Moiré fringes) that emerges from the interaction of the excitation pattern and the sample is used to collect high-frequency information from the image focal plane. In this way, a set of 15 images is typically generated that further requires computational processing (*i.e.*, Fourier transform) using complex algorithms to remove the periodic structure and reconstruct the final high-resolution image.

Compared to other Super-resolution microscopy technologies, SR-SIM does not demand sophisticated sample preparation procedures. However, since usually 15 individual images have to be acquired to obtain a final high-resolution image, SR-SIM increases sample light exposure and imaging time, thereby limiting the ability to capture dynamic processes. The technology works well for thin specimens such as *Drosophila* macrophages and primary spermatocytes in cell culture (Wegel *et al.* 2016), and has been recently adopted for live imaging of microtubule dynamics in *Drosophila* S2 cells (Shao *et al.* 2011) and neuronal transport processes in the *Drosophila* wing (Vagnoni and Bullock 2016). SR-SIM has also been successfully combined with light sheet microscopy to optimize the contrast of in-focus structures in time-lapse recordings of *Drosophila* embryogenesis (Keller *et al.* 2010).

#### STED/RESOLFT:

STimulated Emission Depletion (STED) microscopy (Hell and Wichmann 1994) utilizes two lasers: one excitation laser, and a superimposed, red-shifted, donut-shaped depletion laser to produce images with a lateral resolution of ∼50–80 nm and an axial resolution at the order of 100 nm (Figure 4C). While the excitation laser activates fluorophores in the focal volume, the donut-shaped depletion laser simultaneously returns them back from the excited state to the ground state. As a result, fluorescence signals are only detected from the remaining small focal volume in the center. This demands high laser power and longer laser dwell times than conventional optical sectioning microscopy, enhancing sample bleaching and phototoxic effects. In *Drosophila* research, STED microscopy has successfully been applied to study neuromuscular junctions (Kittel *et al.* 2006; Willig *et al.* 2006), to image planar cell polarity protein complexes at the intercellular junctions of fixed wings (Lau *et al.* 2011), and to visualize vesicle motions in living larvae (Schneider *et al.* 2015).

The principle of STED microscopy has recently been extended by REversible Saturable Optical Linear Fluorescence Transitions (RESOLFT) technology, which facilitates live imaging approaches at light levels that are reduced by up to six orders of magnitude compared to STED microscopy. Using *Drosophila* larvae, RESOLFT has been successfully employed to image the microtubule cytoskeleton by focusing through the intact larval cuticle at nanoscale resolution (Schnorrenberg *et al.* 2016).

#### PALM/STORM:

PALM (Betzig *et al.* 2006; Hess *et al.* 2006) and STORM (Rust *et al.* 2006) are nearly identical single-molecule localization-based Super-resolution microscopy techniques. Both technologies rely on photoswitchable fluorescent dyes or proteins that are, in a first step, stochastically activated by an activation laser applied at low power (Figure 4C). After image acquisition, activated fluorophores are photobleached (PALM) or switched into a reversible dark off-state (STORM) by an inactivation laser. Although hundreds of fluorophores may reside in the same diffraction-limited focal volume, only a subset of them are excited and captured during each activation–inactivation cycle. The final image is then reconstructed by merging all of the detected single-molecule emission events. In this way, PALM and STORM have pushed the resolution limit to a lateral resolution of 20 nm and an axial resolution of 50–60 nm. While PALM was originally published as using photoactivatable or photoconvertible FPs and STORM using synthetic dyes, both types of fluorophores are nowadays generally interchangeable between the two single-molecule localization technologies. Extension of PALM and STORM to multicolor and three-dimensional imaging has further increased its applicability (Shechtman *et al.* 2016).

PALM and STORM require careful probe selection, particularly for multicolor imaging modalities. Nevertheless, the applicability of these nanoscale technologies to *Drosophila* research has been successfully demonstrated by quantitative analysis of intercellular adhesion in the embryo (Truong Quang and Lenne 2015) and the analysis of neuronal compartments in fly brains (Maglione and Sigrist 2013).

## Fluorescence Imaging Applications in *Drosophila* Research

The availability of powerful imaging technologies and the versatility of genetic markers are key drivers for the fast progress in *Drosophila* research. Nearly, any kind of cellular component [*e.g.*, nucleic acids (see *Labeling of nucleic acids*), proteins (see *Labeling of proteins*), organelles (see *Labeling of organelles and other cellular structures*), cell populations (see *Labeling of cells*), organs, and even entire organisms (see *Labeling of organs and organisms to capture morphogenesis*)] can be specifically visualized and even functionally manipulated (see *Controlling the function of cells using optogenetic tools*) with the use of fluorophores.

### Labeling of nucleic acids

Fluorescence *In Situ* Hybridization (FISH) is a cytogenetic technique that uses fluorescently labeled probes that hybridize with the complementary mRNA or DNA sequences in fixed specimens. Multicolor FISH allows the labeling of multiple target sequences in combination, and is generally only limited by the number of available fluorophores with sufficiently separated light spectra. In *Drosophila* research, FISH is a standard method to study the spatiotemporal expression patterns of mRNAs in tissues or to highlight its enrichment in subcellular regions (Lécuyer *et al.* 2008). For example, FISH has been used for mapping of the localization of thousands of mRNA molecules in genome-wide screens in the embryo, ovary, and larval tissues (Lécuyer *et al.* 2007; Jambor *et al.* 2015; Wilk *et al.* 2016). Along with the gene expression data derived from classical nonfluorescence *in situ* hybridization experiments (Tomancak *et al.* 2002, 2007), these annotated patterns are available through publicly accessible databases such as the Berkeley *Drosophila* Genome Prpject, Fly-FISH, and the Dresden Ovary Table.

### Labeling of proteins

As described in the section titled Immunofluorescence, virtually any protein can be visualized by antibody-based IF staining techniques. However, these techniques are not capable of capturing the dynamics of biological systems. With the introduction of GFP (Shimomura *et al.* 1962; Prasher *et al.* 1992), the transgenic expression of FPs quickly became a standard tool to study gene expression; protein localization, dynamics, and function; and tissue morphogenesis by live imaging approaches.

#### Gene expression and protein localization patterns:

In *Drosophila*, cell type- and tissue-specific gene expression patterns were originally explored by enhancer trap screens based on the random integration of transposable elements carrying LacZ (Bier *et al.* 1989), Gal4 (Hayashi *et al.* 2002), or FP (Mollereau *et al.* 2000; Akimoto *et al.* 2005) reporters (see *Expression of genetically encoded FPs*). These reporter genes do not necessarily mimic endogenous expression levels due to different mRNA stability, as well as protein expression and degradation, rates.

Protein trap screens are based on the same methodological concept, but provide both temporal and spatial information as the candidate protein is fused to an FP (see *Expression of genetically encoded FPs*). The great number of available protein trap fly lines has tremendously expanded our knowledge about protein expression and protein localization across different tissues, and most of these image data are publicly available through online databases (Kelso *et al.* 2004; Ryder *et al.* 2009; Knowles-Barley *et al.* 2010).

Alternatively, fusion FPs can also be expressed from ectopic genomic constructs, usually referred to as third-copy alleles (Venken *et al.* 2006; Sarov *et al.* 2016). This approach has been undertaken in studies that analyzed the subcellular localization of Rab-mediated membrane transport processes by expressing FP-tagged Rab versions under the control of a ubiquitous promoter (Marois *et al.* 2006) or using the Gal4/upstream activating sequence (UAS) system (Zhang *et al.* 2007; Chan *et al.* 2011). To control for the possibility that the FP might interfere with the function of the target protein, the fusion protein-bearing transgene needs to be crossed into a null mutant background that is devoid of the corresponding unlabeled protein of interest (known in *Drosophila* as a genetic rescue experiment). However, unphysiological expression levels may distort subcellular protein localization patterns and may interfere with cellular functions. Tagging proteins at their endogenous loci by targeted transgenesis avoids that problem (Kelso *et al.* 2004), and is considered the gold standard in studying physiological gene expression and protein localization patterns (Venken *et al.* 2011).

#### Protein dynamics and protein numbers:

The analysis of protein dynamics and protein numbers is fundamentally based on the ability to perform *in vivo* live imaging in *Drosophila*. One of the most commonly used techniques to assess protein dynamics in *Drosophila* is Fluorescence Recovery After Photobleaching (FRAP). Detailed protocols explaining sample preparation, image acquisition, and image analysis are available (Mavrakis *et al.* 2008; David *et al.* 2012). In a FRAP approach, fusion FPs are illuminated with a laser at sufficiently high power to bleach the fluorophore. A series of images of the region-of-interest is taken before (prebleach) and immediately after FP bleaching (postbleach) at a high frame rate, allowing the extraction of various parameters including the recovery rate and the directionality of recovery to assess protein dynamics. With regard to membrane proteins, the recovery rate provides information about the protein turnover rate, while the directionality of recovery provides information on lateral diffusion dynamics in the lipid bilayer. The capability of FRAP assays has, for example, been demonstrated by a study showing that membrane protein dynamics differ in the anterior and posterior parts of the *Drosophila* embryo (Firmino *et al.* 2013).

Fluorescence loss in photobleaching (FLIP) is another strategy to indirectly measure protein dynamics. FLIP is similar to FRAP and therefore is often performed complementarily (Wilfling *et al.* 2013). In FLIP, photobleaching is repeatedly performed just outside the imaged region-of-interest. The protein dynamics are then assessed from the gradual loss-of-fluorescence that occurs when FP-tagged proteins can diffuse between the bleached and the imaged region. FLIP analyses have, for example, revealed that the movement of mRNAs is restricted within myofibers of *Drosophila* body wall muscle cells (van Gemert *et al.* 2009).

Direct assessment of protein dynamics is enabled by photoactivatable FPs, such as paGFP (Patterson and Lippincott-Schwartz 2002). The photoactivation technique has been adopted in the *Drosophila* field to label protein fusion histones (Post *et al.* 2005), microtubuli (Murray and Saint 2007), cell signaling components (Mavrakis *et al.* 2009), and cell adhesion proteins (Huang *et al.* 2009). Other tools that utilize the photoswitching capability of FP variants have been developed more recently (Chudakov *et al.* 2007). For example, the excitation and emission spectra of the photoswitchable FP Dendra2 undergo a significant red shift upon excitation with blue light, resulting in a switch of emitted photons from the green to the red channel. A study that reports on the use of this tool to study protein dynamics in *Drosophila* has recently been published (Lu *et al.* 2016).

Fluorescence correlation spectroscopy (FCS) is a modern imaging technique that allows the analysis of protein dynamics and the quantification of absolute protein numbers in living cells. In an FCS setup, a laser constantly illuminates a very small diffraction-limited region-of-interest within the part of the cell under study, *e.g.*, the nucleus or cytoplasm, at high temporal resolution. The intensity fluctuations at the detector that occur each time a tagged protein enters, leaves, or passes the three-dimensional (3D) observation volume provide a measure to calculate protein dynamics and absolute protein numbers. In *Drosophila*, FCS has, for example, been employed to study the molecular dynamics of the morphogen DPP during wing development (Wang *et al.* 2004). Furthermore, FCS has enabled the analysis of molecular dynamics of nuclear proteins, *e.g.*, H2B, to monitor changes in the state of chromatin during *Drosophila* embryogenesis (Bhattacharya *et al.* 2009).

#### Protein interactions:

Analysis of protein interactions is another important application of FPs for *in vivo* life imaging in *Drosophila*. One of the most commonly used methods to study protein interactions is based on the electronic energy transfer, termed Förster Resonance Energy Transfer (FRET), between two FP-tagged proteins that are in close proximity.

Importantly, FRET studies can be performed with existing FP constructs commonly used in *Drosophila* research. For FRET, it is crucial that the emission spectrum of the donor FP (*e.g.*, cyan fluorescent protein) sufficiently overlaps with the excitation spectrum of the acceptor FP (*e.g.*, yellow fluorescent protein). When two FP-tagged proteins interact, the excitation of the donor FP results in an efficient energy transfer to the (nonexcited) acceptor FP, leading to a decreased emission of photons by the donor FP (*i.e.*, quenching) and an increased emission of photons by the acceptor FP. Commonly used approaches that indirectly estimate the FRET efficiency as a measure of the distance between interacting proteins are (i) the sensitized emission method (*i.e.*, increase in acceptor emission intensity) and (ii) the acceptor photobleaching method (*i.e.*, increase in donor emission intensity upon acceptor photobleaching). Both methods are compatible with a conventional optical sectioning microscope, but do require a number of internal quality control measures to estimate the photobleaching of the donor and acceptor FPs, or the rate of direct excitation and bleed-through that would lead to a misinterpretation of the signals in the FRET channel. In *Drosophila* research, the classical FRET approach has been used among others to study cell signaling (Lissandron *et al.* 2007), cellular ion levels (Gordon and Dickinson 2006), enzyme activities (Takemoto *et al.* 2007), and cell mechanics/mechanotransduction (Cai *et al.* 2014).

As an alternative to the intensity-based FRET approach, the quenching of the donor FP can be measured using fluorescence lifetime imaging (FLIM), which has recently been employed to study cell mechanics (Eder *et al.* 2017) and viral infection pathways (Smelkinson *et al.* 2017) in *Drosophila*. In FLIM, the contrast of an image does not depend on the emission spectra of the fluorophores but rather on their individual lifetime (*i.e.*, the excited-state decay rate). Hence, in a FLIM-FRET setup, the FRET efficiency can be estimated from the decrease of the fluorescence lifetime of the donor FP due to quenching by the acceptor FP. This time-resolved imaging approach is independent from photobleaching or variable fluorescence intensity, and therefore less susceptible to imaging artifacts. However, it requires expensive secondary microscopy equipment (*e.g.*, a pulsed laser and time-correlated single photon counting (TCSPC) photon-counting electronics).

As an alternative to FRET and FLIM-FRET analyses, BImolecular Fluorescence Complementation allows visualization of protein interactions by complementation between split FP fragments. In such an experimental setup, the coding sequence of the FP is split and each fragment is fused to one of the two proteins of interest. When the two proteins of interest interact, the two FP fragments come in close proximity, reconstitute a functional FP, and emit fluorescence upon excitation. This way, protein interactions have been studied in *Drosophila* adults (Benton *et al.* 2006), larvae (Plaza *et al.* 2008; Gohl *et al.* 2010), and embryos (Hudry *et al.* 2011).

### Labeling of organelles and other cellular structures

The application of fluorescence microscopy-based approaches on cell membranes, organelles, and other subcellular structures serves as important landmarks to determine the intracellular localization of proteins, monitor the subcellular outcomes of functional genomics studies, and track dynamic intracellular transport processes. Specific cell membranes and organelles can be labeled using transgenes encoding FP-tagged signal sequences, protein domains, or entire proteins that are unique for the respective cell or organelle membrane. In *Drosophila*, the plasma membrane can be selectively labeled using FPs fused to the mouse transmembrane protein CD8 (Lee and Luo 1999) or the transmembrane domain of human CD4 protein (Han *et al.* 2011). FP fusions with glycophosphatidylinositol are also targeted to the plasma membrane, with higher levels along the basolateral membrane in epithelial tissues (Greco *et al.* 2001). Alternative membrane targeting motifs include farnesylation, myristoylation, and palmitoylation sequences. A set of different lipid-binding motifs (*e.g.*, Pleckstrin homology (PH) domains) can be used to visualize different cellular phosphoinositide pools (Balla and Várnai 2009). The addition of a nuclear localization sequence to FPs has long been used to efficiently visualize the cell’s nucleus (Davis *et al.* 1995; Shiga *et al.* 1996). Histone fusions are another way to achieve FP targeting to nuclei (Clarkson and Saint 1999; Henikoff *et al.* 2000). A commonly used signal sequence to label membranes of the ER includes the KDEL motif (ER retention sequence) (Snapp *et al.* 2004). The Golgi apparatus can be labeled using an FP fusion with galactosyltransferase (a resident Golgi enzyme) (Snapp *et al.* 2004). Mitochondrial marker transgenes have been generated by FP fusions with the cytochrome *c* oxidase-targeting signal (Cox and Spradling 2003; Pilling *et al.* 2006). The diverse nature of membrane compartments involved in intracellular membrane trafficking can be visualized through specific surface proteins such as the Rab-GTPases (Dunst *et al.* 2015). Other important cellular structures that can be selectively labeled using FP fusions include the actomyosin network (Edwards *et al.* 1997; Royou *et al.* 2004), as well as microtubuli (Grieder *et al.* 2000). Fluorescent dyes that selectively label organelles due to their specific physicochemical properties (*e.g.*, pH and membrane potential) provide a suitable alternative to the use of transgenic constructs. Commonly used dyes to label organelles include LysoTracker, MitoTracker, and ER-Tracker to label lysosomes, mitochondria, and the ER.

### Labeling of cells

The *Drosophila* research community has created a great number of fluorophore-based labeling tools that facilitate the tracing of cell lineages during development, the monitoring of cell migration trajectories, the reconstruction of neuronal projections, and the identification of functional interactions at the cellular level. Cell lineage tracing allows the identification of the entire progeny of a single cell to study signals regulating cell fate decisions in the development of tissues and organs from precursor (stem) cells. Principle requirements for genetically encoded tools to efficiently label cell lineages are control over their temporal and spatial induction, and stable inheritance of the induced state during cell divisions.

The simplest way to label individual cells or cell populations is by expressing a single fluorophore that remains in the cytoplasm, or that is targeted to a specific subcellular compartment (see *Labeling of organelles and other cellular structures*). Fluorophore expression can be temporally and spatially controlled by the enhancer-driven Gal4/UAS system (Brand and Perrimon 1993). Single-color cell labeling has particularly been employed as clonal markers in various mosaic approaches [reviewed in Lee (2014)]. However, single-color labeling does not provide sufficient information for anatomical studies in complex tissues or organs. Here, a diverse spectrum of unique colors to label different populations of cells at the same time would be advantageous. This is enabled by the *Brainbow* technology based on the Cre-Lox recombination system, in which the Cre recombinase drives rearrangements of DNA fragments encoding for a restricted set of different FPs that are flanked by loxP-sites (Livet *et al.* 2007). The stochastic rearrangement process results in a multitude of FP combinations and different hues, facilitating multicolor labeling of cells and their progeny. This versatile genetic paintbrush technology has quickly been adapted to *Drosophila* [see Richier and Salecker (2015) for an excellent review]. In *Drosophila*, *Brainbow*-inspired technologies have been extensively applied in the field of neurobiology (Hadjieconomou *et al.* 2011; Hampel *et al.* 2011), but also in a wide range of nonneuronal cell populations (Förster and Luschnig 2012; Boulina *et al.* 2013; Worley *et al.* 2013; Kanca *et al.* 2014). Here, tissue- or cell type-specific expression of the FP-encoding cassette is typically mediated by the Gal4/UAS system (Brand and Perrimon 1993) in combination with heat-shock promoter-driven expression of the FLP or Cre recombinase. A more detailed review on mosaic analysis techniques in *Drosophila* has been published in the FlyBook compendium (Germani *et al.* 2018).

In addition to lineage tracing, mapping of cell-to-cell interactions is of equal importance. Again, this field of *Drosophila* research has been particularly driven by neurobiologists aiming to reconstruct the connectivity of neural circuits. One such approach to *trans*-synaptically label neuronal connections, termed GFP Reconstitution Across Synaptic Partners (GRASP), was originally developed in *Caenorhabditis elegans* (Feinberg *et al.* 2008) and was immediately adapted to *Drosophila* (Gordon and Scott 2009). GRASP is based on the expression of complementary (nonfunctional) split-GFP fragments on the extracellular membranes of different neuronal populations that, upon synaptic interaction, reconstitute a functional fluorescent GFP reporter. In addition to studying neuronal connections, GRASP has also been used to study cell-to-cell interactions in nonneuronal cell populations of the developing wing (Roy *et al.* 2014).

### Labeling of organs and organisms to capture morphogenesis

In addition to labeling cells to study functional interactions in tissues and organs, the above mentioned fluorophore-based labeling tools have also been successfully employed to capture the morphogenesis, organogenesis, and development of entire organisms. These kinds of studies are typically performed on living animals and require gentle fluorescence microscopy technologies with high acquisition speeds, such as spinning disk or light sheet microscopes (see *Optical sectioning microscopy*). Imaging and tracking of morphogenetic movements further requires robust cellular landmarks that can be automatically detected by image analysis algorithms. Hence, nuclear markers such as histone-coupled or membrane-coupled FPs (see *Labeling of organelles and other cellular structures*) are preferentially used. In this way, all of *Drosophila* embryogenesis (Krzic *et al.* 2012; Tomer *et al.* 2012), as well as the morphogenesis of the pupal thorax (Bosveld *et al.* 2012) and wing (Aigouy *et al.* 2010; Etournay *et al.* 2015), have already been visualized in 3D image stacks from which the movement of each individual cell has been extracted by means of computational image analysis [Etournay *et al.* (2016) and reviewed in Keller (2013)].

### Controlling the function of cells using optogenetic tools

Optogenetic tools deploy the expression of light-sensitive proteins to manipulate the physiological state of cells using laser light at specific wavelengths in a temporally precise manner. In *Drosophila* research, the field of optogenetics is gaining increasing attention and has successfully been applied to control neuronal activity by the expression of light-gated variants of the cation channel channelrhodopsin-2 (ChR2). As an example, optogenetic activation of specific populations of dopamine-releasing neurons in combination with an odor stimulus converged into an artificial light-induced memory (Riemensperger *et al.* 2016). Optogenetics has further been used to study the experience dependency of the male fly courtship behavior (Inagaki *et al.* 2014).

Apart from its broad use in neuroscience, the applicability of optogenetics to cardiac research in *Drosophila* has recently been explored. To replace the need for electrical stimulation and its inherent side effects, expression of an optogenetically controlled ChR2 in fly hearts has been used to directly control heart rhythm by stimulating its pacing (Alex *et al.* 2015). Other research areas include, for example, the artificial optogenetic regulation of gene expression patterns (Chan *et al.* 2015), signaling pathways (Kaur *et al.* 2017), and morphogenesis (Guglielmi *et al.* 2015) at high spatiotemporal resolution to examine developmental processes in the fly. A more detailed review on optogenetic techniques in *Drosophila* has been published in the FlyBook compendium (Simpson and Looger 2018).

## Concluding Remarks

The combination of *Drosophila* genetic and reverse genetics toolkits with modern imaging approaches will undoubtedly keep fruit fly research at the forefront of modern biology. Over the 100 years of the existence of the field, fly geneticists have accumulated an impressive array of genetic tools designed to label RNA, proteins, cellular compartments, and whole cells. These meticulously maintained and broadly shared resources are increasingly complemented by systematically generated transgenic reagent sets. Together, we are well on the way to having suitable fluorescent protein fusions for every gene in the genome. Moreover, the advanced genome engineering toolkit allows the targeting of these reagents to any fly tissue, with precise control over the timing and spatial restriction of the expression domain. The reagent sets are not only comprehensive but are often built as versatile platforms capable of accepting new developments in the dynamically moving science of genetically encoded fluorescent reporters.

**Figure 5:**
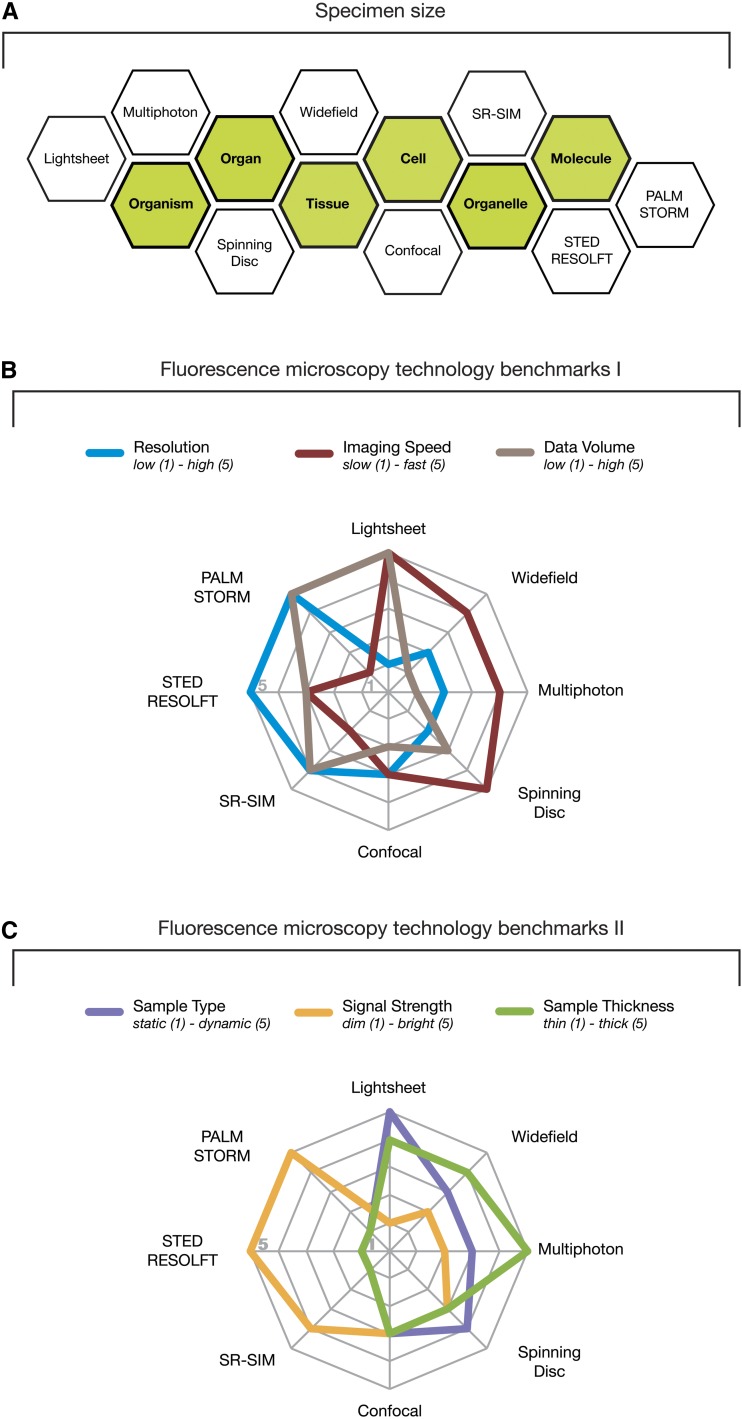
Capabilities and limitations of fluorescence microscopy technologies. (A) A comprehensive set of fluorescence imaging technologies allow the visualization of specimens across multiple orders of magnitude in size, ranging from molecules to entire organisms. (B and C) Each individual fluorescence microscopy technology has its inherent capabilities and limitations with regard to the resolution of the final image, the imaging speed, the volume of generated data, the type and thickness of the sample, and the strength of the signal. For each fluorescence microscopy technology, these benchmark criteria are illustrated in two radar diagrams ranging from 1 (center) to 5 (margin) to facilitate comparison between imaging technologies and aid the selection of a suitable imaging technology that fulfills the experimental needs. PALM, Photo-Activated Localization Microscopy; RESOLFT, REversible Saturable Optical Linear Fluorescence Transitions; SR-SIM, Superresolution Structured Illumination Microscopy; STED, STimulated Emission Depletion; STORM, STochastic Optical Reconstruction Microscopy.

Thomas Morgan could not possibly have imagined the type of visualizations of the *Drosophila* life cycle that we have at hand today, and if he were he may have opted for an organism that is developing at a slower pace, is more transparent, and less protected from the environment. *Drosophila* is certainly far from an ideal model organism for imaging, but the challenges of imaging the fast gastrulation of *Drosophila*, and the need to penetrate deep inside living larvae to reach relevant tissues and to monitor the activities of brains in animals that are very capable of flying away, have pushed the development of microscopy technology to greater speeds, depths, and more creative sample-mounting paradigms. Furthermore, the availability of versatile genetic tools has ensured that most, if not all, new imaging modalities are optimized or outright developed for applications in the *Drosophila* research field. Available multidimensional imaging technologies bridge the scales from organisms to molecules across multiple orders of magnitude in size (Figure 5A). *Drosophila* research newcomers willing to engage in this fascinating expedition toward the visualization of the unknown will have to carefully consider which type of microscopy technology suits their experimental demands. However, each microscopy technology has its specific capabilities and individual limitations (Figure 5, B and C).

Evaluating the synergy of *Drosophila* and imaging research fields from yet another angle; contemporary *Drosophila* research is so advanced that it is no longer sufficient to look at one gene in a fixed preparation of a fly tissue. Drosophilists aspire to capture biological processes in flies that are live and in totality, following thousands of cells and other labeled components throughout entire developmental stages of the animal. Even a single one of these recordings creates data sets that are far beyond the ability of human observers to comprehend, necessitating the application of sophisticated computer algorithms to extract quantitative information from vast multidimensional images. Here, *Drosophila* provides some of the most challenging and comprehensive data, on which modern machine learning approaches will have to prove their worth. In addition, since the interaction of experts with the data will remain indispensable if we are to gain biological insights, *Drosophila* research will even push such distinctly nonbiological fields as 3D data visualization and immersive virtual reality for data exploration and annotation.

With the boundless ambition of *Drosophila* researchers to, for example, monitor and manipulate entire neuronal circuits in freely behaving adult flies, we imagine that, in the future, the imaging field will have to develop laser-generated holograms that are projected into flies’ brains to simultaneously manipulate the physiological state of multiple neurons and entire neuronal networks at once to enhance existing neuronal circuit maps controlling animal behavior. Clearly, for future generations of *Drosophila* researchers, there lie exciting times ahead.
